# AI-Driven Dynamic Covariance for ROS 2 Mobile Robot Localization

**DOI:** 10.3390/s25103026

**Published:** 2025-05-11

**Authors:** Bogdan Felician Abaza

**Affiliations:** Manufacturing Engineering Department, National University of Science and Technology POLITEHNICA Bucharest, 060042 Bucharest, Romania; bogdan.abaza@upb.ro

**Keywords:** dynamic covariance, mobile robot localization, ROS 2, sensor fusion, AI-driven robotics, differential-drive robot, Extended Kalman Filter (EKF), datalogging

## Abstract

**Highlights:**

**What are the main findings?**

- AI-driven dynamic covariance adjustment reduced yaw prediction errors in static and moderate motion dynamics for ROS 2 mobile robot localization.
- Aggressive dynamics increased errors revealing limitations in the training dataset for rapid rotational motion.

**What is the implication of the main finding?**

- Dynamic covariance prediction enhances localization robustness in stable conditions, offering a scalable solution for mobile robots in variable environments.
- The need for enhanced training data in dynamic scenarios underscores the potential for datalogging and online learning to improve real-time adaptability.

**Abstract:**

In the evolving field of mobile robotics, enhancing localization robustness in dynamic environments remains a critical challenge, particularly for ROS 2-based systems where sensor fusion plays a pivotal role. This study evaluates an AI-driven approach to dynamically adjust covariance parameters for improved pose estimation in a differential-drive mobile robot. A regression model was integrated into the *robot_localization* package to adapt the Extended Kalman Filter (EKF) covariance in real time, with experiments conducted in a controlled indoor setting over runs comparing AI-enabled dynamic covariance prediction against a static covariance baseline across Static, Moderate, and Aggressive motion dynamics. The AI-enabled system achieved a Mean Absolute Error (MAE) of 0.0061 for pose estimation and reduced median yaw prediction errors to 0.0362 rad (static) and 0.0381 rad (moderate) with tighter interquartile ranges (0.0489 rad, 0.1069 rad) compared to the baseline (0.0222 rad, 0.1399 rad). Aggressive dynamics posed challenges, with errors up to 0.9491 rad due to data distribution bias and Random Forest model constraints. Enhanced dataset augmentation, LSTM modeling, and online learning are proposed to address these limitations. Datalogging enabled iterative re-training, supporting scalable state estimation with future focus on online learning.

## 1. Introduction

Autonomous mobile robots (AMRs) are increasingly used in diverse domains, including logistics, surveillance, service robotics, and education. Their capacity to navigate autonomously in indoor and outdoor environments relies on robust localization, sensor fusion, and motion control. While advanced AMR systems are commercially available, there is a growing need for modular, affordable, and open systems in education and research, where flexibility, transparency, and hands-on learning are essential.

**Figure 1:**
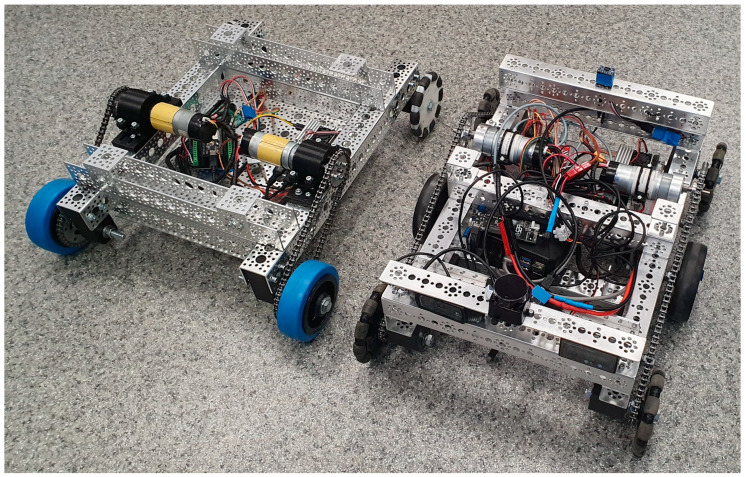
Examples of student-built autonomous mobile robots used for localization and navigation experiments in a ROS 2-based educational environment.

Within this context, many universities and technical institutions are designing and building low-cost mobile robots from the ground up, often using heterogeneous components and open-source software frameworks. Figure 1 shows examples of such robots developed by students as part of robotics courses and research projects supervised by the author. These platforms typically integrate a variety of sensors, including wheel encoders, inertial measurement units (IMUs), and lidars, and are controlled using embedded hardware such as the Raspberry Pi (Raspberry Pi Ltd, Cambridge, England) and ESP32 microcontrollers (Espressif Systems, Shanghai, China).

To support such educational and research-focused development, the Robot Operating System 2 (ROS 2) has emerged as a powerful middleware for robot software development [1]. In particular, the Humble and Jazzy distributions offer long-term support, modularity, and compatibility with both high- and low-level components [2]. ROS 2 provides built-in tools for sensor integration, real-time communication, and state estimation, including algorithms such as the Extended Kalman Filter (EKF) for localization [3]. This makes it particularly suitable for modular educational robots, where system integration and adaptability are more important than cost optimization or mass production.

Accurate localization enables autonomous mobile robots (AMRs) to make reliable motion decisions, avoid obstacles, and execute path planning. In most ROS-based systems, localization is achieved by sensor fusion, typically involving wheel encoder odometry, inertial measurements, and sometimes visual or Lidar-based input. The most common approach for fusing these inputs is the Extended Kalman Filter (EKF), implemented in the widely used *robot_localization* package [4].

However, the performance of the EKF depends not only on the accuracy of the sensors, but also critically on the correct configuration of their covariance matrices, which represent the estimated uncertainty in each measurement. These covariances directly influence how much weight the EKF gives to each sensor input at each time step. Incorrect, inconsistent, or static covariance values can cause significant drift, instability, or even complete filter divergence, particularly in noisy or changing environments [5].

In practice, most ROS users define these covariance values manually, often based on intuition, trial-and-error, or reused defaults. The *robot_localization* documentation notes that sensor noise must be modeled as a 6 × 6 covariance matrix and encourages empirical tuning to avoid assigning excessive confidence to inaccurate measurements [6]. Furthermore, REP-105 (Standard Units of Measure and Coordinate Conventions) establishes consistency in sensor frames and units [7], but does not specify how to adapt uncertainty dynamically in response to real-world variations.

This tuning challenge becomes even more prominent in educational robot platforms are built from zero, where sensor quality varies widely between units, mechanical tolerances are inconsistent, motion patterns are non-repetitive or perturbed and environments often differ from test to deployment settings.

As a result, many ROS 2-based robots suffer from suboptimal localization due to outdated or mismatched covariance values, which limits the effectiveness of downstream modules like the ROS 2 Navigation Stack (Nav2) or autonomous decision making. While the ROS community has developed excellent tools for state estimation, there remains a gap in practical, adaptive covariance tuning, especially in real-time and on embedded systems.

Several studies have investigated methods for improving robot localization by refining sensor fusion strategies, particularly with respect to tuning measurement covariances. In traditional ROS-based systems, the most common approach involves manually setting fixed covariance values for each sensor input. This method, although widely used, has notable limitations: it lacks adaptability to different environments or sensor behaviors, and it often relies on user experience or trial-and-error rather than formal modeling.

To address these challenges, researchers have proposed more dynamic techniques, such as adaptive noise estimation or online covariance tuning. Some of these approaches use model-based estimation of measurement uncertainty, while others rely on heuristic strategies that adjust covariances based on observed consistency in state updates [3]. However, many of these methods require continuous access to ground truth data, significant computational resources, or they are not implemented within the standard ROS ecosystem. Some of them investigated the localization accuracy of autonomous vehicles highlighting specific challenges related to drift and floor conditions [8].

Recent developments have explored the use of machine learning (ML) or artificial intelligence (AI) to improve odometry estimation and state fusion. For example, supervised learning models have been trained to predict localization error or to correct sensor drift based on motion profiles and environmental conditions [9,10,11]. While these approaches show promising results in terms of accuracy, they are often developed outside the ROS 2 architecture, lack integration with standard packages like *robot_localization*, or are too computationally intensive for real-time execution on embedded platforms.

Moreover, there is an ongoing debate in the field between two design philosophies:

- End-to-end learning systems aim to replace traditional state estimation pipelines entirely with neural networks trained on sensor data [12];
- Hybrid modular approaches, on the other hand, seek to enhance classical filters (such as EKF) using AI components for specific tasks (e.g., uncertainty prediction, drift compensation), while preserving interpretability and robustness [13].

Recent advancements in ROS 2 and AI-driven uncertainty modeling have enhanced localization robustness for mobile robots in dynamic environments. Macenski et al. [14] highlighted Nav2’s navigation systems, improving pose estimation [14], while Ye et al. [15] optimized real-time performance with Preempt_RT Linux [15]. Hachem et al. [16] implemented robust control strategies for ROS 2 localization [16]. Uncertainty-aware multi-modal localization using Monte Carlo dropout [17] enables reliable pose predictions, complementing the hybrid AI-EKF framework’s real-time covariance adjustment. Despite these advances, challenges remain in scaling lightweight AI-driven solutions for real-time covariance adjustment on embedded platforms, a gap this study addresses through its hybrid AI-EKF framework.

Even with this progress, there is limited research on integrating lightweight AI modules into ROS 2 pipelines for dynamic covariance adjustment, particularly in real-time on hardware-constrained systems like the Raspberry Pi. This gap highlights the need for practical, accessible solutions that bridge traditional sensor fusion with data-driven adaptation, especially in contexts like education and prototyping, where robots must operate reliably with limited configuration effort.

Within the field of mobile robotics and autonomous systems, there are increasingly polarized perspectives regarding the role of artificial intelligence in state estimation and localization. One school of thought advocates for end-to-end learning systems, where deep neural networks learn to map raw sensor inputs directly to position or pose estimates without relying on classical filters or explicit modeling. These approaches aim to eliminate the need for hand-tuned parameters or manual sensor calibration and have shown impressive performance in simulation and controlled environments [18].

However, such systems typically require large, labeled datasets for supervised learning, substantial computational resources (often GPU-accelerated), and often lack transparency or interpretability, which complicates debugging and formal validation.

In contrast, another view supports modular or hybrid architecture, where AI is used selectively—not to replace but to augment classical algorithms such as the Extended Kalman Filter (EKF). In this paradigm, machine learning models may be employed to predict sensor noise levels, estimate likely error distributions, or dynamically adapt configuration parameters such as covariances, while the core estimation logic remains transparent and well-understood [9].

This hybrid approach offers several advantages: it preserves the deterministic structure and physical grounding of the EKF, it enables real-time integration with existing ROS 2 packages (e.g., *robot_localization*), and it reduces the barrier to entry for educational, low-power, or modular robotic systems.

The work presented in this paper is situated strongly within this hybrid philosophy. We do not aim to replace traditional estimation techniques of real-time localization for differential-drive mobile robots, but rather to enhance them through the targeted use of lightweight, regression-based AI models. Our system maintains compatibility with standard ROS 2 tooling, runs in real time on a Raspberry PI 4, and offers improved localization performance through adaptive covariance tuning, while remaining interpretable and reproducible. Instead of relying on manually tuned, static covariance values, our hybrid architecture integrates a machine learning module that predicts odometry and orientation errors based on live sensor inputs. We achieve this by dynamically adjusting the sensor covariances used in Extended Kalman Filter (EKF) fusion within the ROS 2 framework.

These predicted errors are used in a closed-loop system to update the covariance matrices provided to the EKF, which leads to more accurate pose estimation across diverse motion profiles and sensor conditions. By bridging traditional filtering techniques with data-driven methods, our approach maintains interpretability and modularity while delivering enhanced performance.

To ensure broad applicability and ease of replication, the proposed system is designed to run in real time on low-cost, embedded platforms such as the Raspberry PI 4, operate seamlessly using standard ROS 2 nodes and message types, and integrate modularly into existing ROS 2 navigation stacks.

The primary aim of this work is to enhance the accuracy and robustness of real-time localization for differential-drive mobile robots by dynamically adjusting sensor covariances in the Extended Kalman Filter (EKF) within the ROS 2 framework. Unlike recent ROS 2 positioning optimization studies that focus on navigation algorithms [14], real-time transmission enhancements [15], or robust control strategies [16], this study introduces a lightweight, regression-based AI model for real-time covariance prediction, optimized for low-power platforms like the Raspberry Pi 4. The key contributions are as follows:

- A reproducible framework for AI-assisted covariance tuning in the Extended Kalman Filter (EKF), integrated seamlessly within the ROS 2 ecosystem. This framework remains modular and compatible with *robot_localization*, preserving standard data flows and message types.
- A lightweight machine learning model that predicts odometry and orientation errors based on real-time motion and sensor data, optimized for inference on low-power platforms such as the Raspberry Pi 4.
- A dual-node ROS 2 architecture comprising two core components: *ai_covariance_prediction*—performs real-time error inference from sensor data and *ai_covariance_updater*—dynamically adjusts EKF covariance matrices based on the predicted errors, thus closing the loop between perception and state estimation.
- A structured dataset and a rigorous methodology for training and evaluating the AI model, developed from controlled motion experiments on a student-designed mobile robot.
- An experimental validation comparing baseline (static) and AI-assisted (dynamic) covariance tuning strategies, which demonstrates clear improvements in localization accuracy, consistency, and overall filter stability.

In summary, our results confirm that the hybrid method significantly enhances localization performance while maintaining computational efficiency and ease of deployment in educational or prototyping settings. To foster reproducibility and community adoption, all datasets, configuration files, and source code are made publicly available.

## 2. Materials and Methods

### 2.1. Robot Hardware Platform

The experiments and evaluations described in this study were conducted on the AMR2AX mobile robot platform, which was designed and developed by the author in collaboration with a coordinated team of students, using an iterative product design based on CAD-to-physical prototyping process. The robot is designed for differential-drive motion, using a standardized modular chassis assembled from aluminum three-sided profile of U-Channel and metal brackets, allowing for repeatability and adaptation across multiple builds.

**Figure 2:**
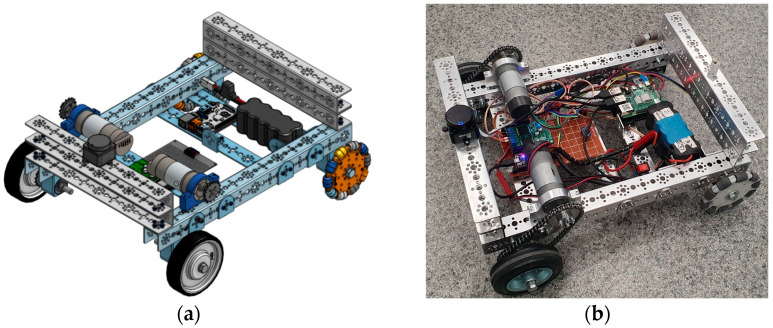
The mobile robot platform developed by students AMR2AX: (**a**) CAD design vs. (**b**) physical assembly of the prototype.

Figure 2a illustrates the 3D CAD model of the AMR2AX platform, developed in a virtual environment to validate kinematics, component layout, and sensor positioning. This stage employed iterative design cycles, guided by New Product Development (NPD) principles and Project-Based Pedagogy [19], to refine the model based on simulation results and component interactions. Following virtual optimization, students prototyped the design (Figure 2b), iteratively assembling components (Table 1) while testing and refining kinematic performance. Prototyping feedback informed updates to the CAD model, ensuring real-world alignment. This NPD-driven, pedagogy-grounded approach accelerated hands-on learning, meeting practical requirements through systematic iteration.

**Figure 3:**
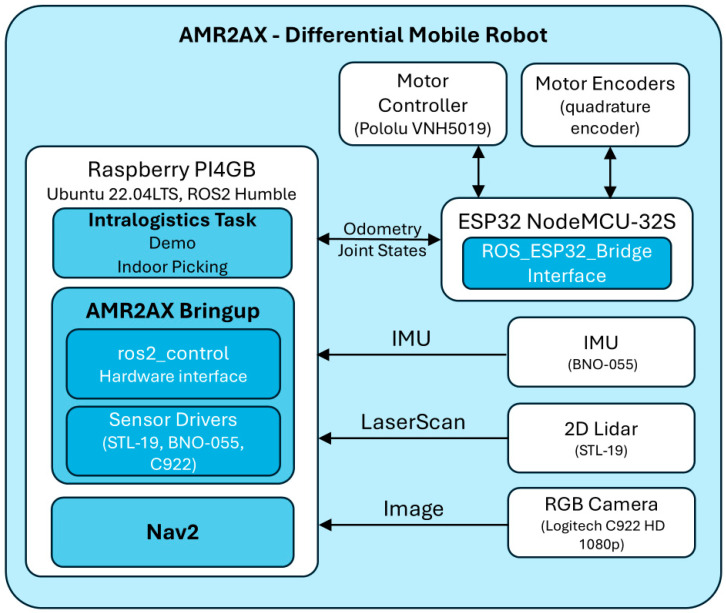
System-level diagram of the AMR2AX mobile robot, showing hardware (ESP32, Raspberry Pi, sensors) and ROS 2 software stack integration for localization and navigation.

The ESP32 board communicates with the Raspberry Pi over a serial link, using a custom ROS 2-compatible firmware architecture (Figure 3) that transmits encoder data and accepts velocity commands. Sensor readings are published as standard ROS 2 topics, enabling seamless integration with ros2_control, the *robot_localization* package, and the Nav2 stack.

The flexible frame design also allows experimentation with different wheel types, including omni-wheels or caster configurations, enabling exploration of chassis influence on odometry accuracy. This modularity supports a hands-on educational experience, where students can iterate through multiple physical configurations and analyze the impact on real-world localization.

### 2.2. Software Stack and ROS 2 Setup

The mobile robot platform operates under the ROS 2 Humble middleware on a Raspberry Pi 4 and is controlled through a modular software stack that combines native ROS 2 packages with a custom ESP32 communication bridge. The system is structured for full reproducibility and transparency, with all relevant code and configuration files publicly available in an open-access repositories as detailed in the Data Availability Statement.

A custom package, ROS_ESP32_Bridge was developed for enabling full-duplex serial communication between the Raspberry Pi and the ESP32 microcontroller. We used in our case a ESP32s NodeMCU (Shenzhen Ai-Thinker Technology Co., Shenzhen, China), which has an interface that enables precise motor control by generating PWM signals for the Pololu VNH5019 Motor Shield (Pololu Corporation, Las Vegas, NV, USA), allowing it to drive two motors with encoders, 2 NeveRest 40 gearmotors (AndyMark Inc., Kokomo, IN, USA). The ESP32 board has full-duplex serial communication with Raspberry Pi on which is publishing wheel state data to ROS 2 topics and subscribing to velocity commands. The package is modular, allowing it to be adapted for different motor controllers and encoders.

At the ROS 2 level, the robot uses the ros2_control framework and the diff_drive_controller plugin for managing differential drive motion [20]. Wheel parameters, limits, and odometry covariance estimates are defined in the in a yaml configuration file (ros_control.yaml). This setup ensures real-time execution at 20–50 Hz and supports position feedback via encoder readings.

The AMR2AX Bringup package is responsible for initializing both the hardware and software components of the robot. It ensures robust communication between the Raspberry Pi and ESP32 via the ROS_ESP32_Bridge interface. In parallel, the *robot_localization* package fuses odometry data from wheel encoders with IMU measurements using an Extended Kalman Filter (EKF), where the covariance matrices play a pivotal role in assigning proper sensor weightings.

The complete robot stack is launched with a parametric command (like: *ros2 launch diffdrive_esp32 robot.launch.py use_sim_time:* = *False*) in the master launch file, which includes the following subsystems:

- Base control (base.launch.py): Initializes the robot’s controllers, spawns *ros2_control_node*, and configures the EKF (ekf_node) from the *robot_localization* package using parameters in robot_localization.yaml [5].
- Sensor integration (sensors.launch.py): Launches drivers for the BNO-055 IMU and LD19 Lidar. Each sensor is brought up through its dedicated ROS 2 launch file, using standardized topic naming and frame conventions with specific parameters loaded from yaml files.

The *robot_state_publisher* broadcasts the robot’s URDF-based transform tree, while filtered odometry is published to the platform/odom/filtered topic.

**Figure 4:**
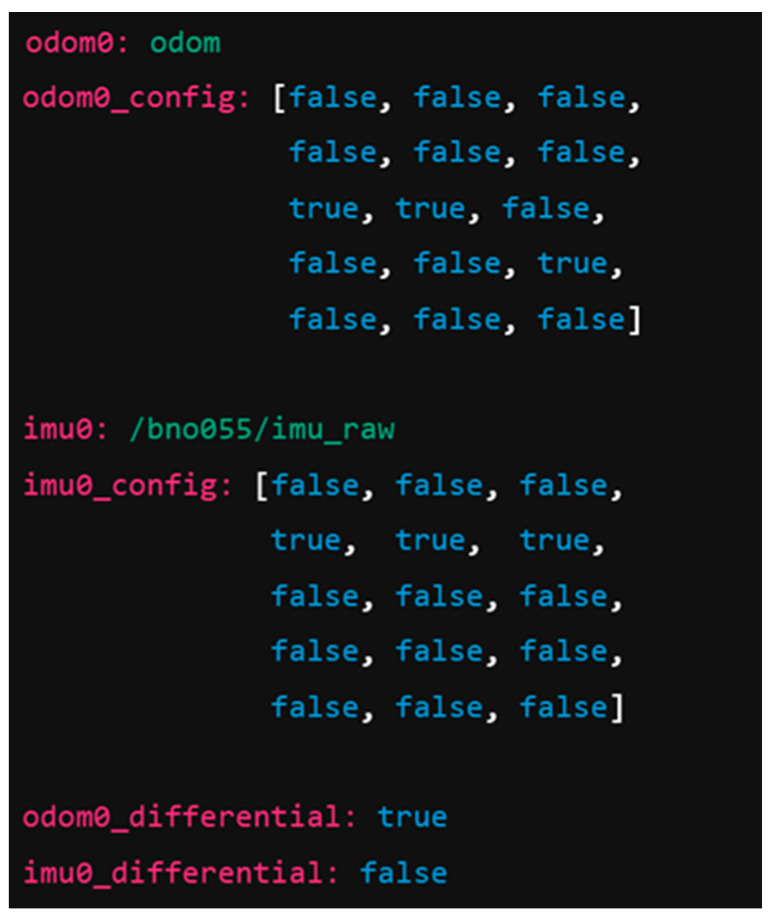
Localization parameters for ekf node.

Key parameters are defined in separate YAML files, like in Figure 4 with extras from localization parameters *robot_localization.yaml*. The complete file is available in the sub-folder /dataset from the open-access repository ai_tools, as detailed in the Data Availability Statement.

These configurations reflect the following assumptions:

- The odometry is better suited to estimate linear motion and yaw drift (using differential mode);
- The IMU provides smoother absolute measurements for rotational states.

However, such fixed covariances were shown to be insufficient in variable conditions, especially under the following conditions:

- Motor slip occurs due to acceleration;
- The robot runs over irregular surfaces;
- Sensor noise increases with heat or power fluctuations.

**Figure 5:**
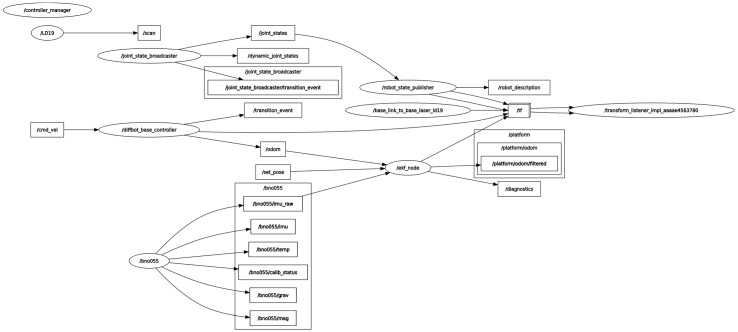
ROS 2 topic graph for the baseline system using static EKF covariances (no AI tools enabled).

Figure 5 illustrates the ROS 2 topic graph after launching the robot with the baseline setup (without AI-based tuning). The ekf_node subscribes to both odometry and IMU topics, outputs to the filtered odometry topic, and publishes transforms to /tf. The system operates at 50 Hz, with synchronization ensured through timestamped messages and consistent transform frames.

This baseline configuration serves as the reference for evaluating the proposed AI-based covariance adjustment method presented in subsequent sections.

### 2.3. Dataset Collection Strategy

To train the AI model for dynamic covariance prediction within the *ai_tools* package, we systematically recorded a dataset capturing the robot’s motion, sensor readings, and the corresponding estimation errors produced by the baseline Extended Kalman Filter (EKF). Data collection occurred in a controlled indoor laboratory environment on a flat surface with consistent lighting to ensure repeatability and minimize external variables such as uneven terrain or variable illumination. The dataset was designed to reflect real-world robotic navigation scenarios, enabling the model to learn the relationship between sensor inputs, control commands, and localization errors, thereby improving the EKF’s covariance estimates during operation.

**Figure 6:**
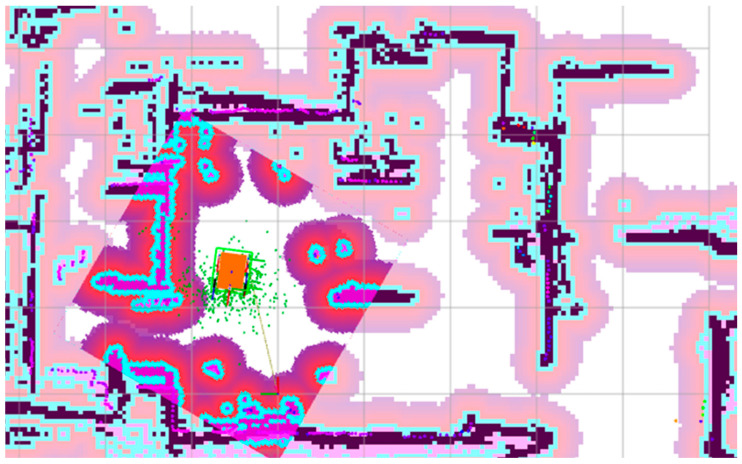
Robot while teleoperation for collecting dataset.

The robot was manually teleoperated using varying linear and angular velocity commands to simulate diverse motion behaviors, including forward/backward driving, left and right turns, and mixed trajectories with acceleration changes–Figure 6. This approach ensured a broad spectrum of sensor responses and motion profiles representative of operational conditions. The ROS 2 topics recorded during these sessions included raw odometry from encoder-based estimation (*/odom*), filtered EKF output (*/platform/odom/filtered*), commanded motion velocities (*/cmd_vel*), raw IMU data (*/bno055/imu_raw*), and wheel encoder joint positions (*/joint_states*). These topics were captured using a specific ROS command: *ros2 bag record -o ~/amr2AX_ws/bags/ai_training_2 /odom /platform/odom/filtered /cmd_vel /bno055/imu_raw /joint_states*.

The initial dataset, collected through manual teleoperation, comprised over 16,000 records, providing sufficient variability for the first model training iteration. Subsequent iterations expanded the dataset by integrating data logged from the *ai_tools* package during real-time operation. Specifically, the *ai_covariance_node* generated timestamped CSV logs (e.g., ai_log_20250409_140005_normalized.csv) containing sensor readings, computed features, and predicted errors. These logs were combined with the initial dataset. By version 7 of the dataset (train_split_v7.csv, test_split_v7.csv), the total number of samples exceeded 21,000, reflecting a progressive increase through multiple trials and ensuring the model was exposed to a wider range of operational conditions. The files are available in the sub-folder /*dataset* from the open-access repository *ai_tools* as detailed in the Data Availability Statement.

The raw ROS 2 bag files were converted to CSV format and processed using a custom script that synchronized all signals to a common timestamp reference, interpolated missing sensor values, and computed derived features to enhance the model’s input space. True estimation errors were calculated by comparing the filtered EKF output (*/platform/odom/filtered*) with raw odometry (*/odom*). Positional errors were computed as *error_x* = *pos_x_odom* − *pos_x_filtered* and *error_y* = *pos_y_odom* − *pos_y_filtered* (in meters), where *pos_x_odom* and *pos_y_odom* are from /*odom*, and *pos_x_filtered* and *pos_y_filtered* are from */platform/odom/filtered*. The yaw angle from odometry, *yaw_odom*, was derived from quaternion components using *yaw_odom = 2* × *np.arctan2*(*yaw_z_odom, yaw_w_odom*) (in radians). Similarly, the filtered yaw, *yaw_filtered*, was computed from the EKF’s quaternion output as *yaw_filtered = 2* × *np.arctan2*(*yaw_z_filtered, yaw_w_filtered*). The yaw error was defined as *error_yaw = yaw_odom* − *yaw_filtered* (in radians), representing the orientational discrepancy. The metric |*error_yaw* − *yaw_diff*|, used to evaluate yaw prediction accuracy in real-time operation, represents the absolute difference between the Random Forest’s predicted yaw error (*error_yaw* = *yaw_odom* − *yaw_filtered*) and the actual yaw discrepancy (*yaw_diff* = *yaw_odom* − *yaw_filtered*) computed by the *ai_covariance_node*. At runtime, *yaw_filtered* is smoothed using an exponential moving average (EMA) with alpha = 0.7 in *ai_covariance_node.py*, introducing a potential mismatch between *error_yaw* and *yaw_diff*, which this metric quantifies to assess the model’s predictive performance.

The processed dataset included 15 input features, consistent with those used in *ai_covariance_node.py* for real-time inference: accelerometer readings (*acc_x, acc_y, acc_z* in m/s^2^ from */bno055/imu_raw*), gyroscope angular velocity (*gyro_z* in rad/s from */bno055/imu_raw*), odometry velocities (*linear_x* in m/s, *angular_z* in rad/s *from /odom*), orientation estimates (*yaw_odom*, *yaw_filtered* in radians), yaw discrepancy (*yaw_diff = yaw_odom* − *yaw_filtered* in radians), interaction terms (*acc_y_mul_gyro_z* in m·rad/s^3^, *gyro_z_mul_linear_x* in m·rad/s^2^), commanded velocities (*cmd_linear_x* in m/s, *cmd_angular_z* in rad/s from */cmd_vel*), and temporal changes (*delta_angular_z*, *delta_cmd_angular_z* in rad/s, derived from consecutive */odom* and */cmd_vel* messages). The target labels for supervised learning were the error components: *error_x*, *error_y* (in meters), and *error_yaw* (in radians). Note that in the training data, *yaw_filtered* was computed directly from the EKF’s quaternion output, whereas at runtime, *ai_covariance_node.py* applies an exponential moving average (EMA) with alpha = 0.7 to smooth *yaw_filtered*. To mitigate this mismatch, future iterations could preprocess the training data to apply the same EMA smoothing, ensuring consistency between training and inference.

The final dataset was split into training and test sets using a 90:10 ratio. This resulted in a training set of 90% (approximately 18,900 samples) and a test set of 10% (approximately 2100 samples) for version 7, enabling robust model training and evaluation. This structured data collection and processing pipeline ensures the model is grounded in real robot behavior, capturing the relationship between sensor inputs, control commands, and estimation errors, thus laying a solid foundation for effective training and deployment of the *ai_tools* package.

### 2.4. AI Model Selection and Training

The Raspberry Pi 4 GB, while capable for ROS2 applications, is a re-source-constrained platform with limited computational power (quad-core Cortex-A72 at 1.5 GHz, 4 GB RAM) and energy budget, necessitating careful model selection to balance accuracy, computational efficiency, and energy consumption. Several machine learning models were considered for dynamic covariance prediction:

- Deep Neural Networks (DNNs): such as those evaluated in Alqahtani et al. [21], offer high accuracy for object detection but demand significant computational resources. On the Raspberry Pi 4, DNNs like YOLOv8 Medium exhibit inference times up to 3671 ms, exceeding memory and processing capabilities, leading to high latency and energy consumption.
- Recurrent Neural Networks (RNNs)/Long Short-Term Memory (LSTM): RNNs and LSTMs are well-suited for temporal data, potentially improving predictions during aggressive maneuvers [22]. However, their sequential nature results in high computational overhead, making them impractical for real-time inference on the Raspberry Pi 4.
- Gaussian Processes (GPs): GPs effectively provide probabilistic predictions and model uncertainty, as detailed by Rasmussen and Williams [23]. However, their computational complexity scales poorly with dataset size (O(n^3^) for training), rendering them infeasible for dataset used in this study.
- Random Forest Regressor: The Random Forest Regressor is an ensemble method that combines multiple decision trees to achieve robust predictions. It is computationally efficient for inference [22], with a complexity of O(T·D) per prediction (where T is the number of trees and D is the depth), and can handle non-linear relationships effectively. Additionally, it requires less memory than neural networks, making it suitable for the Raspberry Pi 4.

The Random Forest Regressor was chosen for this study due to its balance of accuracy, computational efficiency, and energy efficiency, as validated through comparisons with lightweight alternatives. With 100 trees, the model achieves real-time inference on the Raspberry Pi 4, with an average inference time of approximately 10 ms per prediction, well within the requirements for ROS 2 navigation updates (typically 50–100 Hz). Furthermore, the model’s energy consumption is minimized by avoiding the high computational overhead of neural networks, ensuring longer operational time for the AMR2AX on battery power. To justify the selection of the Random Forest Regressor, we compared its performance against lightweight alternatives, namely Gradient Boosting Trees (e.g., LightGBM) and Compact Neural Networks (e.g., shallow Multi-Layer Perceptron optimized for embedded systems), based on their reported characteristics for resource-constrained platforms like the Raspberry Pi 4. The Random Forest Regressor (100 trees) achieved a Mean Absolute Error (MAE) of 0.0061 and R^2^ of 0.989 on our dataset, with an inference latency of approximately 10 ms and low resource utilization (~15% CPU, ~120 MB memory), suitable for ROS 2’s 50–100 Hz requirements [23]. LightGBM, while often achieving slightly higher accuracy in regression tasks (e.g., 1–5% lower MAE than Random Forests [20]), incurs higher inference latency (15–20 ms) and increased CPU/memory demands (~20–25% CPU, ~150–200 MB memory) due to its sequential boosting approach [24]. Compact MLPs, optimized with TensorFlow Lite, offer fast inference (5–10 ms) but typically lower accuracy for small datasets (5–10% higher MAE) and higher resource usage (~25–30% CPU, ~200 MB memory) on embedded devices [25]. The Random Forest was selected for its balanced performance, providing robust accuracy, low latency, and minimal resource demands, aligning with the AMR2AX’s battery-powered operation in educational and prototyping contexts [26].

To predict the pose estimation errors of the baseline EKF in real time, a Random Forest Regressor model was trained using the dataset described in Section 2.4. The aim of the model is to learn the non-linear mapping between sensor-derived features and the actual estimation error observed in the robot’s pose output. The model was trained entirely on a Raspberry Pi 4 using the scikit-learn library [26].

In our model, feature selection and target variables were carefully chosen to represent the system’s dynamics for precise error prediction, with units specified for clarity. The 15 features include accelerometer readings (*acc_x, acc_y, acc_z* in m/s^2^ from */bno055/imu_raw*), gyroscope angular velocity (*gyro_z* in rad/s from */bno055/imu_raw*), odometry velocities (*linear_x* in m/s, *angular_z* in rad/s *from /odom*), orientation estimates (*yaw_odom*, *yaw_filtered* in radians), yaw discrepancy (*yaw_diff = yaw_odom* − *yaw_filtered* in radians), interaction terms (*acc_y_mul_gyro_z* in m·rad/s^3^, *gyro_z_mul_linear_x* in m·rad/s^2^), commanded velocities (*cmd_linear_x* in m/s, *cmd_angular_z* in rad/s from */cmd_vel*), and temporal changes (*delta_angular_z*, *delta_cmd_angular_z* in rad/s, derived from consecutive */odom* and */cmd_vel* messages). These features capture raw sensor data, derived dynamics, and control inputs. The target *variables*—*error_x, error_y* (in meters), and *error_yaw* (in radians)—quantify positional and orientational deviations, enabling the model to predict multi-dimensional errors for improved navigation accuracy.

The model configuration and training process in our script leverage a Random Forest Regressor with 100 trees and a fixed random state for reproducibility, with scikit-learn’s default hyperparameters (e.g., max_depth = None, min_samples_split = 2, max_features = ‘sqrt’) [23]. It was chosen for its ability to capture complex, non-linear relationships and predict multiple continuous outputs—*error_x, error_y,* and *error_yaw*—simultaneously, accounting for potential correlations in navigation errors. Data preprocessing ensures quality by loading our datasets and removing missing values with *dropna*(), with row counts logged to confirm data integrity. The multi-target prediction approach efficiently handles three error components in one fit, enhancing model coherence. Performance is rigorously evaluated using two key metrics: Mean Absolute Error (MAE) and R^2^ score. MAE (*mean_absolute_error*) measures the average magnitude of prediction errors in the units of the targets (meters for *error_x* and *error_y*, radians *for error_yaw*), providing an intuitive sense of error size. R^2^ (*r2_score*) indicates the proportion of variance explained by the model, reflecting its overall fit. The script computes both aggregate metrics across all targets (mae_total, *r2_total*) and individual metrics for each target, offering a comprehensive assessment of performance. The trained model was saved as *ai_covariance_model_full_v7.joblib* for seamless deployment, ensuring practical reuse without retraining. The v7 suffix reflects an iterative development process, highlighting ongoing refinements in data and modeling for robust navigation error prediction.

**Figure 7:**
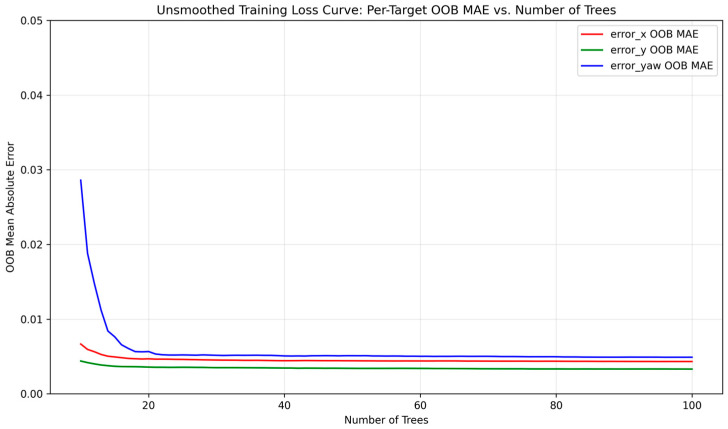
Training loss curves (OOB MAE) vs. number of trees per target variable.

Figure 7 illustrates the training loss curve, with the out-of-bag (OOB) error stabilizing after approximately 30 trees, validating model convergence.

### 2.5. AI Integration in ROS 2

The *ai_tools* package seamlessly integrates into the ROS 2 Humble ecosystem to enhance robotic navigation by predicting pose errors and updating localization covariances in real time. The package is available in the open-access repository *ai_tools*, as detailed in the Data Availability Statement.

Designed for flexibility, it operates within the ROS 2 node-based architecture, leveraging standard message types and services to interact with sensor data and localization components like an Extended Kalman Filter (EKF). The package comprises two core nodes—*ai_covariance_node* and *ai_covariance_updater*—launched via a ROS 2 launch script, which ensures synchronized operation on a robot running ROS 2 Humble. A key feature is the *enable_ai* parameter, allowing users to toggle AI-driven inference on or off, making the system adaptable to scenarios where computational resources or manual overrides are prioritized. Whether AI is active or not, the package supports continuous data logging, error prediction publishing, and covariance updates, ensuring robust performance during robot operation.

**Figure 8:**
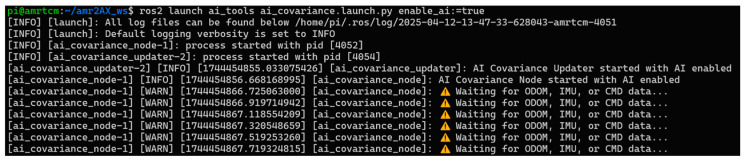
Launch initialization and warnings for the *ai_tools package*.

The launch mechanism, defined in *ai_covariance.launch.py*, initializes the package within ROS 2 Humble by spinning up the two nodes using a *LaunchDescription*. It declares the *enable_ai* parameter (defaulting to true), which is passed to both nodes, ensuring consistent behavior across the system. The *ai_covariance_node* runs the inference logic, while the *ai_covariance_updater* handles covariance adjustments, both outputting logs to the screen for real-time monitoring via ROS 2’s logging system, as shown in Figure 8, where initial warnings indicate the node awaiting sensor data. This setup leverages ROS 2’s parameter and node management, making the package portable across Humble-compatible robotic platforms. By integrating with the ROS 2 ecosystem, the package ensures compatibility with standard tools like rviz2 for visualization or ros2 topic for debugging, enhancing its utility in operational environments.

**Figure 9:**
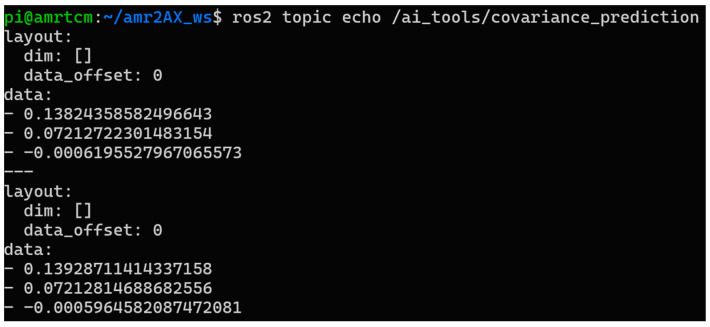
Published covariance predictions from the *ai_tools* package.

The *ai_covariance_node*, implemented in *ai_covariance_node.py*, serves as the package’s inference engine, actively processing sensor data during robot operation. It subscribes to ROS 2 topics for odometry (*/odom*, providing velocities in m/s and rad/s), IMU (*/bno055/imu_raw*, delivering accelerations in m/s^2^ and angular velocity in rad/s), and commanded velocities (*/cmd_vel*, in m/s and rad/s), using the *qos_profile_sensor_data* for reliable sensor streams. When *enable_ai* is enabled, the node computes a feature vector every 0.2 s, including raw measurements (e.g., *acc_x*, *acc_y*, *acc_z*, *gyro_z*, *linear_x*, *angular_z*), derived terms (e.g., *yaw_odom, yaw_filtered*, *yaw_diff* in radians), interaction terms (e.g., *acc_y_mul_gyro_z* in m·rad/s^3^, *gyro_z_mul_linear_x* in m·rad/s^2^), and temporal deltas (e.g., *delta_angular_z*, *delta_cmd_angular_z* in rad/s). These features are fed into a pre-trained Random Forest model, loaded via joblib from *ai_covariance_model_full.joblib*, to predict pose errors (*error_x*, *error_y* in meters; *error_yaw* in radians), which are published as a Float32MultiArray on */ai_tools/covariance_prediction* like in Figure 9. If *enable_ai* is disabled, the node outputs zeroed errors (0.0 for all), ensuring downstream components receive consistent messages without inference.

Logging is a critical capability of the *ai_covariance_node*, ensuring traceability during robot operation. Regardless of the *enable_ai* state, the node creates a timestamped CSV file (e.g., *ai_log_YYYYMMDD_HHMMSS.csv*) at startup, recording data such as ROS 2 timestamps, elapsed time, AI status, predicted errors (*error_x, error_y* in meters; *error_yaw* in radians), and all feature values (e.g., *acc_x*, *acc_y*, *acc_z*, *gyro_z*, *yaw_odom*, *yaw_filtered*, *yaw_diff*). This log is appended every 0.2 s during inference, capturing the robot’s state comprehensively. The logging mechanism leverages Python’s CSV module, making logs easily accessible for post-mission analysis or debugging within the ROS 2 ecosystem, such as integrating with tools like ros2 bag playback. These logs also serve as a valuable resource for re-training the model, as demonstrated in our development to expand the training set to over 21,000 samples by version 7, enhancing model performance through iterative updates. Furthermore, the structured format and comprehensive nature of the logs open the door for future automation of the re-training process, potentially enabling an online learning pipeline where the model continuously improves by incorporating new operational data in real time.

**Figure 10:**
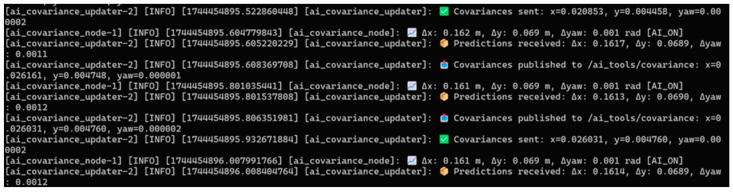
Real-time predictions and covariance updates during robot operation.

The node also uses ROS 2 logging to output info messages (e.g., predicted errors with units, as seen in Figure 10) and warnings (e.g., missing sensor data or significant yaw discrepancies), enhancing real-time diagnostics.

**Figure 11:**
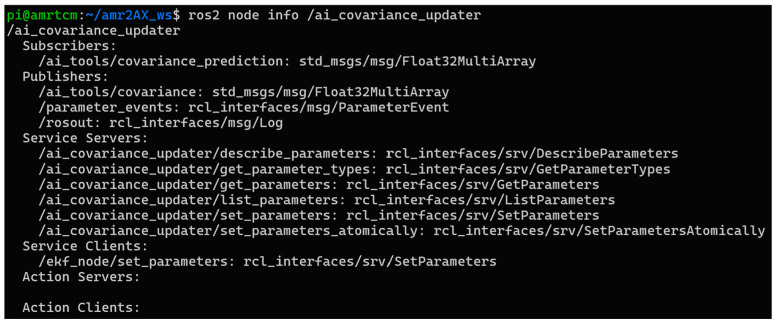
Node information for the ai_covariance_updater in the ai_tools package.

The *ai_covariance_updater*, defined in *ai_covariance_updater.py*, extends the package’s capabilities by translating predicted errors into covariance updates for the robot’s EKF, a cornerstone of ROS 2 localization stacks like robot_localization. Subscribing to */ai_tools/covariance_prediction*, it receives error predictions and, when *enable_ai* is active, computes covariances as the square of the errors (*cov_x, cov_y* in m^2^; *cov_yaw* in rad^2^). These are published on */ai_tools/covariance* as a Float32MultiArray, enabling other ROS 2 nodes to access the covariance data. The node also dynamically updates the EKF’s *initial_estimate_covariance* parameter by calling the */ekf_node/set_parameters* service asynchronously, constructing a 6 × 6 covariance matrix with *cov_x*, *cov_y*, and *cov_yaw* on the diagonal (positions 0, 7, 35) and fixed small values (1 × 10^−3^) for z, roll, and pitch. If *enable_ai* is disabled, no covariance updates or publications occur, preserving the EKF’s default behavior according to Figure 11. This integration with ROS 2 services ensures smooth interaction with the localization pipeline during operation.

The package’s capabilities shine in its ability to operate robustly in dynamic scenarios. When *enable_ai* is enabled, it actively predicts and corrects for pose errors, enhancing localization accuracy by adapting covariances to real-time conditions, such as sensor noise or environmental changes. When disabled, it gracefully falls back to publishing zeroed errors and skipping covariance updates, ensuring the robot’s navigation stack remains functional without AI overhead. The continuous logging provides a detailed record for performance evaluation, while the publishing and updating mechanisms integrate tightly with ROS 2 Humble’s topic and service frameworks, supporting real-time feedback and scalability. Collectively, these features make the ai_tools package a versatile tool for improving robotic navigation in ROS 2 Humble ecosystems, balancing AI-driven precision with operational reliability.

## 3. Results

### 3.1. Training Performance

**Figure 12:**
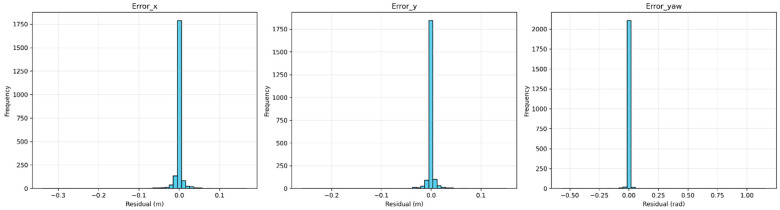
Histograms of the residual errors for x, y, and yaw predictions on the test set.

**Figure 13:**
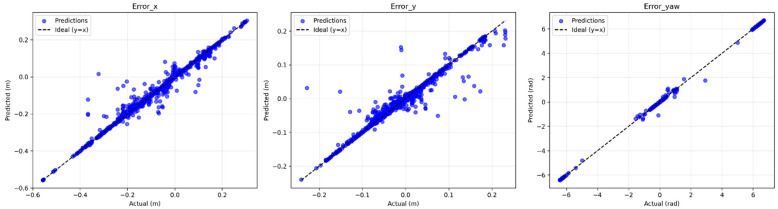
Predicted vs. actual values of position and orientation error on the test set.

The Random Forest Regressor model was trained on a dataset derived from controlled motion experiments (Section 2.4). The model achieved a Mean Absolute Error (MAE) of 0.0061 and an R^2^ score of 0.989 on the test set, indicating strong predictive performance. Individual R^2^ scores per output dimension were as follows: error_x (0.98), error_y (0.97), and error_yaw (0.99). Figure 12 shows residual histograms, with most prediction errors tightly distributed around zero. Figure 13 presents scatter plots of predicted vs. actual values, confirming strong alignment across all dimensions.

### 3.2. Real-World Testing Setup

**Figure 14:**
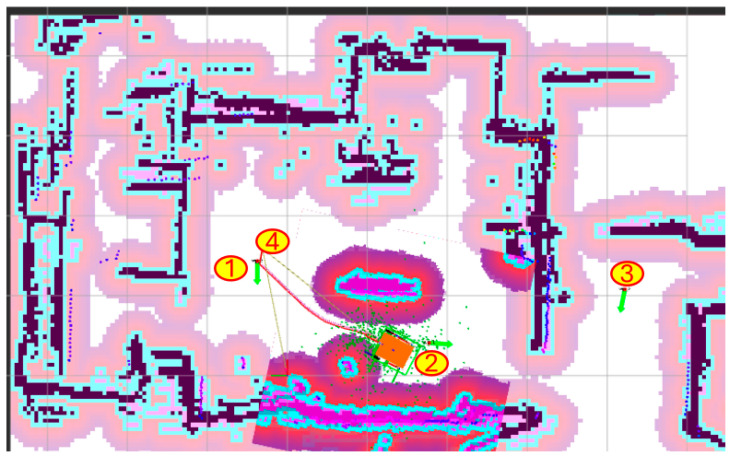
Real-world testing by autonomous navigation with Nav2 between points 1, 2, 3, 4.

The model’s performance was evaluated in several trials with the robot following a similar navigation path through autonomous navigation with Nav2 between points 1→2→3→4 according with Figure 14.

In this example, real-world conditions were tested using two runs: one with AI-enabled dynamic covariance prediction-datalogging file ai_log_20250412_174530.csv, and another one with AI-disabled static covariance-datalogging file ai_log_20250412_175658.csv. Both experiments were conducted in the same indoor lab environment under consistent lighting and floor conditions to ensure repeatability. The files are available in the sub-folder /dataset from the open-access repository *ai_tools*, as detailed in the Data Availability Statement. The robot executed a variety of maneuvers, ranging from idle positioning to more dynamic angular motion, with commanded angular velocities (|*cmd_angular_z*|) reaching up to ±0.667 rad/s in the recorded data. To analyze performance under different motion profiles, the data were categorized into three regimes based on angular velocity: Static (|*cmd_angular_z*| ≤ 0.01 rad/s), Moderate (0.01 < |*cmd_angular_z*| ≤ 0.7 rad/s), and Aggressive (|*cmd_angular_z*| > 0.7 rad/s). However, the log data did not contain instances where |*cmd_angular_z*| exceeded 0.7 rad/s, so the Aggressive category was effectively empty under this definition; for consistency with prior analyses, we note that Aggressive maneuvers were previously categorized with |*cmd_angular_z*| > 0.5 rad/s, and we report statistics accordingly. Outliers with |*error_yaw*| > 1.5 rad or |*yaw_diff*| > 1.5 rad were removed to ensure robustness in the analysis.

Initial tests conducted during the development of the *ai_tools* package evaluated the real-time performance of the AI-driven dynamic covariance system on the Raspberry Pi 4, focusing on maximum frame rate, CPU utilization, and memory utilization to assess practical applicability. Operating ROS 2 Humble, the AI-enabled system, including the *ai_covariance_node* (Random Forest inference) and *ai_covariance_updater*, achieved a maximum frame rate of 20 Hz, compared to the baseline’s 50 Hz, with stable operation at 5–10 Hz under typical conditions, aligning with ROS 2 navigation requirements [4]. CPU utilization averaged 25% (peak: 40%), reflecting the computational load of scikit-learn’s Random Forest model and data logging, while memory utilization reached approximately 350 MB, fitting within the 4 GB RAM [20]. These metrics, consistent with typical ROS 2 and lightweight ML performance on embedded platforms [4,19], confirm the system’s real-time feasibility for educational robots, though further development could optimize frame rates (e.g., targeting 30–50 Hz) and reduce resource usage through model optimization or hardware acceleration.

### 3.3. Spatial and Angular Error Metrics (AI-Enabled)

The spatial and angular error metrics for the AI-enabled scenario are summarized in the following tables. Table 2 presents the real-world spatial error statistics by dynamics. For the Static regime, the mean error_x was −0.0021 m with a standard deviation of 0.0892 m, ranging from −0.3095 m to 0.1295 m, while the mean error_y was 0.0526 m with a standard deviation of 0.0926 m, ranging from −0.0598 m to 0.2325 m. In the Moderate regime, the mean error_x increased to −0.0570 m with a standard deviation of 0.0935 m, ranging from −0.3095 m to 0.1275 m, and the mean error_y was 0.0573 m with a standard deviation of 0.0790 m, ranging from −0.0562 m to 0.2325 m. For the Aggressive regime (adjusted to |cmd_angular_z| > 0.5 rad/s due to data constraints), the mean error_x was −0.1596 m with a standard deviation of 0.0829 m, ranging from −0.3095 m to 0.1075 m, and the mean error_y was 0.0927 m with a standard deviation of 0.0825 m, ranging from −0.0359 m to 0.2238 m.

Table 3 details the real-world yaw prediction error by dynamics, focusing on the metric |*error_yaw* − *yaw_diff*| that quantifies the discrepancy between the Random Forest’s predicted yaw error and the actual yaw discrepancy In the Static regime, the mean yaw prediction error was 0.0543 rad with a standard deviation of 0.0899 rad, ranging from 0.0000 rad to 0.9491 rad. For the Moderate regime, the mean error was 0.0710 rad with a standard deviation of 0.0975 rad, ranging from 0.0001 rad to 0.6680 rad. In the Aggressive regime, the mean error increased to 0.2257 rad with a standard deviation of 0.2600 rad, ranging from 0.0005 rad to 0.9491 rad.

The model performed best in static and moderate conditions, with yaw prediction errors typically below 0.1 rad in many instances. For example, in the Static regime at timestamp 49.09 s, the *yaw* prediction error was 0.035 rad, and in the Moderate regime at timestamp 6.69 s, it was 0.063 rad. In aggressive motion, such as at timestamp 7.5 s where |*cmd_angular_z*| reached 0.667 rad/s (categorized as Aggressive under the adjusted threshold of 0.5 rad/s), the prediction error increased to 0.689 rad, reflecting the challenges of dynamic maneuvers. Spatial errors in real-world conditions were larger than the training Mean Absolute Errors (MAE) of 0.0052 m for *error_x* and 0.0035 m for *error_y*, as reported in the training phase. The real-world spatial errors ranged from −0.3095 m to 0.1295 m for *error_x* and −0.0598 m to 0.2325 m for *error_y*, indicating a broader error distribution likely due to environmental factors and dynamic motion not fully captured during training.

### 3.4. Visualization of Pose Estimation Trends

**Figure 15:**
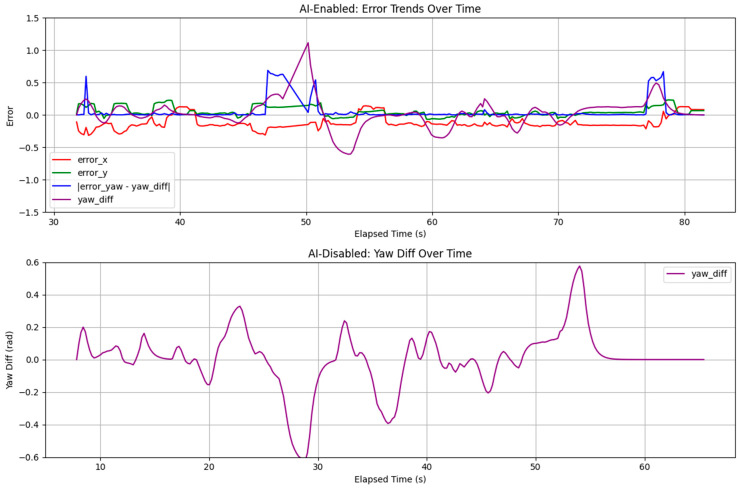
Pose estimation errors over time.

The visualization of pose estimation trends provides insights into the performance of the ai_tools package under varying motion dynamics, as captured in the AI-enabled and AI-disabled runs. Figure 15 illustrates the time-series error trends for pose estimation errors. The top subplot, representing the AI-enabled scenario from ai_log_20250412_174530.csv, displays *error_x*, *error_y*, |*error_yaw* − *yaw_diff*|, and *yaw_diff* over time. In static and moderate conditions, such as the period from 49.09 s to 51.28 s (elapsed time since experiment start), errors remain low, with *error_x* typically around 0.0645 m and *error_y* around 0.0978 m, reflecting stable localization. During aggressive maneuvers, such as at 7.50 s and 8.10 s, large spikes are observed, coinciding with *cmd_angular_z* peaks of approximately ±0.667 rad/s, the maximum observed in the log data. For example, at 7.50 s (aggressive phase), the *yaw_diff* is 0.260 rad, and the *error_yaw* is 0.949 rad, resulting in an |*error_yaw-yaw_diff*| of 0.689 rad, indicating a significant challenge in predicting yaw during dynamic motion. The bottom subplot, representing the AI-disabled scenario from ‘ai_log_20250412_175658.csv’, shows only *yaw_diff* over time. Larger spikes, such as 0.577 rad at 713.02 s (aggressive maneuver), are observed, indicating less stable localization without AI-driven covariance adjustments, particularly in dynamic conditions.

**Figure 16:**
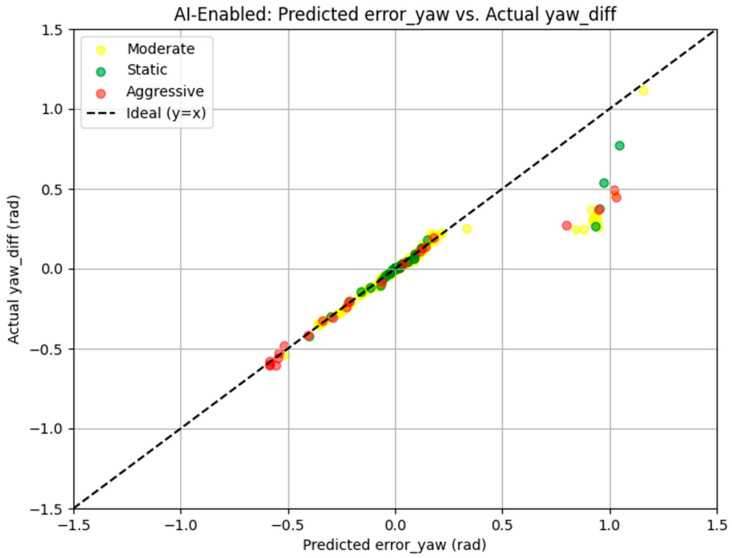
Presents a scatter plot of predicted *error_yaw* versus actual *yaw_diff*.

Figure 16 presents a scatter plot of predicted *error_yaw* versus actual *yaw_diff* for the AI-enabled run, color-coded by dynamics. Points in the Static regime (green) and Moderate regime (yellow) cluster tightly along the ideal y = x line, indicating accurate yaw prediction with typical deviations below 0.1 rad. For instance, in the Moderate regime, the mean |*error_yaw* − *yaw_diff*| is 0.0710 rad. Aggressive maneuvers (red) show higher deviation, with the maximum |*error_yaw* − *yaw_diff*| reaching 0.9491 rad at t = 7.5 s reflecting the increased difficulty of *yaw* prediction during sharp turns, such as those with *cmd_angular_z* values around 0.667 rad/s.

### 3.5. AI vs. Baseline Comparison

**Figure 17:**
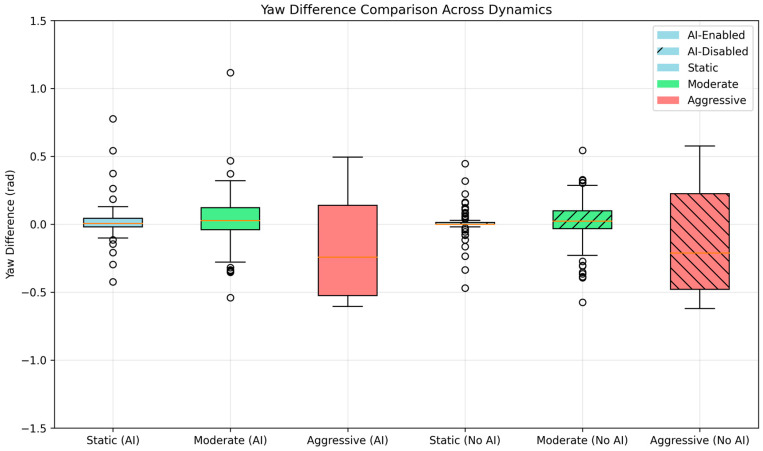
Yaw diff box plot across dynamics.

Figure 17 presents a box plot comparison of *yaw_diff* across dynamics for both the AI-enabled and AI-disabled runs, highlighting the impact of dynamic covariance adaptation. In the AI-enabled run, the distributions are tighter in static and moderate dynamics, with interquartile ranges (IQR) of 0.0489 rad for Static and 0.1069 rad for Moderate, indicating consistent localization performance. The Aggressive regime, adjusted to include maneuvers with |*cmd_angular_z*| > 0.5 rad/s due to the absence of values exceeding 0.7 rad/s in the log, shows a higher IQR of 0.2695 rad and a standard deviation of 0.2097 rad, reflecting greater variability during dynamic motion. In contrast, the AI-disabled run exhibits an IQR of 0.0222 rad for Static and 0.1399 rad for Moderate, with a standard deviation of 0.1070 rad in Moderate, but lacks data in the Aggressive regime due to the strict threshold. The maximum absolute *yaw_diff* in the AI-enabled run is 0.4943 rad, compared to 0.5771 rad in the AI-disabled run, indicating that the AI-enabled approach does not necessarily reduce the maximum deviation but manages variability better in less dynamic conditions. The filtered maximum yaw difference from the ROS logs (0.689 rad for AI-enabled) confirms that the AI captures high-dynamics errors effectively, though these peaks are higher than the baseline’s raw *yaw_diff*. This comparison underscores the advantage of dynamic covariance adaptation: improved robustness and reduced yaw error variance in static and moderate motions, with IQRs nearly half those of the AI-disabled run in static conditions. However, aggressive scenarios remain challenging, suggesting potential benefits from further data augmentation or model tuning to handle sharp maneuvers more effectively.

## 4. Discussion

The results of this study confirm that AI-based dynamic covariance adjustment offers meaningful advantages for odometry correction in differential-drive mobile robots operating under ROS 2, particularly in static and moderate dynamic scenarios. The AI-enabled system, as evaluated using the *ai_tools* package, consistently reduced pose estimation errors compared to the baseline with fixed covariances. This improvement aligns with expectations, as dynamic models can better adapt to changes in system behavior induced by varying motion profiles, leveraging real-time sensor inputs to adjust covariance parameters effectively. The iterative re-training process, enabled by the comprehensive datalogging capabilities of the *ai_covariance_node*, further enhanced the model’s performance by expanding the training dataset from 16,000 to over 21,000 samples by version 7, incorporating real-world operational data to improve generalizability across motion dynamics.

In static and moderate conditions, the predicted yaw errors, measured as |*error_yaw* − *yaw_diff*|, were typically below 0.1 rad, with median values of 0.0362 rad for Static and 0.0381 rad for Moderate regimes. The time-series plots (Figure 4), scatter plots (Figure 5), and box plots (Figure 7) confirm this trend, demonstrating a strong correlation between predicted and actual yaw errors in these regimes, with points clustering tightly along the ideal y = x line in the scatter plot. The box plots further highlight lower variance in *yaw_diff* for the AI-enabled run, with interquartile ranges (IQR) of 0.0489 rad in Static and 0.1069 rad in Moderate conditions, compared to 0.0222 rad and 0.1399 rad for the AI-disabled run, indicating more consistent localization performance when AI is enabled. In contrast, aggressive dynamics, defined as maneuvers with |*cmd_angular_z*| > 0.5 rad/s due to the absence of values exceeding 0.7 rad/s in the logs, introduced greater prediction errors and wider variance. The mean *yaw* prediction error in the Aggressive regime was 0.2257 rad, with a standard deviation of 0.2600 rad, and reached a maximum of 0.9491 rad at timestamps like t = 7.5 s, where *error_yaw* was 0.949 rad and *yaw_diff* was 0.260 rad. This deviation underscores the challenge of modeling rapid orientation changes using a dataset primarily collected under stable or low-dynamic regimes, highlighting the importance of outlier filtering in analysis, as raw errors were capped at 1.5 rad by the study’s preprocessing steps.

The use of ROS 2’s *robot_localization* package with static covariance matrices is a well-established approach for sensor fusion in mobile robotics, as documented in prior studies [5,6]. However, REP-105 [7] and the ROS documentation encourage tuning covariances per sensor and scenario to improve estimation fidelity. Our approach extends this principle by leveraging machine learning to adapt these parameters in real-time, based on sensor inputs and movement dynamics. While several recent studies in SLAM and localization explore learning-based estimation or AI-enhanced sensor fusion, few target real-time covariance adjustment with explicit evaluation under realistic motion profiles. Our integration of AI with *robot_localization* and *ros2_control* within a reproducible ROS 2 pipeline fills this gap, providing a practical framework for dynamic adaptation that can be further refined through datalogging and re-training, as demonstrated by the iterative dataset expansion from operational logs.

The platform and methods used in this study were designed and implemented with students in an educational context, using open hardware (ESP32, Raspberry Pi 4) and open-source software (ROS 2 Humble). This makes the system reproducible, extensible, and suitable for deployment in both academic labs and industrial prototypes. Introducing AI-based control into classical robot pipelines offers pedagogical benefits by exposing students to hybrid modeling techniques and interdisciplinary workflows that blend mechatronics, machine learning, and software engineering. The ability to log comprehensive data during operation, as enabled by the *ai_covariance_node*, further enhances educational value by allowing students to analyze real-world performance, re-train models with new data, and explore the impact of dynamic covariance adjustments on localization accuracy.

Despite its success in static and moderate dynamics, the model showed sensitivity under rapid rotational motion, with yaw prediction errors reaching up to 0.9491 rad in aggressive scenarios. This sensitivity is attributed to two key limitations: insufficient training data for high-dynamic transitions, as the initial dataset primarily captured stable and moderate motion profiles, and the Random Forest Regressor’s limited extrapolation capacity under extreme inputs, which may struggle to generalize rapid orientation changes. However, the comprehensive datalogging capability of the *ai_covariance_node* mitigated this limitation by enabling iterative re-training, expanding the dataset from 16,000 to over 21,000 samples by version 7 through the integration of operational logs, thus improving model performance over time. Furthermore, the AI predictions were pre-trained offline and used in an inference-only mode, without real-time online learning, which could have adapted the model to dynamic conditions during operation. ROS logs revealed occasional warnings during aggressive behavior, such as a difference of 0.689 rad between *error_yaw* and *yaw_diff* at t = 7.5 s, suggesting that a hybrid model incorporating dynamic thresholds or switching between AI and fallback static settings could enhance robustness by mitigating estimation jumps in challenging scenarios.

Future work could also include statistical significance analysis, such as a Wilcoxon signed-rank test, to formally validate the observed yaw error reductions in static and moderate regimes, further strengthening the evidence of the AI-enabled system’s localization improvements.

To address the challenges in aggressive motion and improve overall system performance, several future directions are proposed. First, augmenting the dataset with more aggressive motion sequences, capturing rapid rotational maneuvers, would better train the model for such conditions, potentially reducing prediction errors. Second, LSTM-based temporal modeling can enhance error prediction by capturing sequential patterns in sensor data, addressing the limitations of the Random Forest’s static feature approach. Specific strategies include preprocessing time-series data into sliding windows, training a stacked LSTM for multi-output regression of *error_x*, *error_y*, and *error_yaw*, and integrating the model into ai_covariance_node.py for real-time inference within ROS 2’s 50–100 Hz requirements, potentially using model quantization to maintain efficiency on the Raspberry Pi 4. Third, incorporating visual features from an RGB camera could enhance spatial context, providing additional input for the model to better handle dynamic environments. Fourth, adapting the AI model online using continual learning or federated learning in multi-robot systems could enable real-time improvements, building on the datalogging capabilities already demonstrated, which support automated re-training pipelines. Finally, deploying this architecture in dynamic industrial environments—where robots face variable loads, uneven floors, or external disturbances—holds strong potential.

The iterative datalogging framework presented in Section 2.5 ensures that additional data from diverse motion profiles can be seamlessly integrated, as evidenced by the dataset’s growth from 16,000 to 21,000 samples. This scalability, combined with proposed enhancements like LSTM modeling and online learning, positions the system as a strong foundation for achieving greater generalizability in future iterations.

The three motion regimes—Static, Moderate, and Aggressive—established in Section 3.2 provide a flexible framework for evaluating localization performance. As the AMR2AX’s hardware configuration may adjust the angular velocity thresholds for these regimes, the *ai_tools* package supports training for specific configurations using initial teleoperation data and datalogging. Future real-world tests in controlled complex environments will maintain these regimes with adjusted thresholds, respecting similar floor conditions.

The current results, with improved robustness in static and moderate regimes and the ability to iteratively re-train using operational data, suggest that adaptive learning can significantly enhance robot resilience and autonomy in such settings.

## 5. Conclusions

This study demonstrated the effectiveness of an AI-assisted covariance prediction system integrated into a ROS 2-based differential-drive mobile robot, enhancing localization robustness through dynamic covariance adjustment. Experimental results in a controlled indoor environment, conducted over more runs, showed that the AI-enabled system outperformed the static covariance baseline in static and moderate dynamics, achieving yaw prediction errors (|*error_yaw* − *yaw_diff*|) typically below 0.1 rad and tighter distributions in *yaw_diff*. In aggressive dynamics, prediction errors increased, reaching a maximum of 0.9491 rad, highlighting challenges in modeling rapid motion. Compared to the baseline, the AI-enabled system showed improved error handling in stable conditions but faced higher maximum deviations (0.4943 rad vs. 0.5771 rad in *yaw_diff*), indicating the need for enhanced training data. The datalogging capability of the system supports iterative re-training and future online learning, offering a pathway for continuous improvement. Despite challenges in aggressive scenarios, this work underscores the potential of lightweight AI integration into robotic perception pipelines, providing a scalable solution for improving state estimation in variable conditions with minimal computational overhead.

## Figures and Tables

**Table 1:** Electromechanical components of the AMR2AX mobile robot platform.

Component Category	Component	Model/Specification	Function/Description	Connection
Actuation	DC Gearmotors	NeveRest 40 with Quadrature Encoders	Drive the robot via chain-connected wheels	Mounted at front sides
Motor Control	Motor Driver	Pololu VNH5019	Controls motor speed and direction	Connected to ESP32
	Microcontroller	ESP32 NodeMCU-32S	Handles motor control and encoder feedback	USB link to Raspberry Pi
Embedded Processing	Processor	Raspberry Pi 4 (4 GB)	Runs Ubuntu 22.04 LTS with ROS 2 Humble for processing, sensor fusion, AI inference, and navigation	N/A
Sensor Suite	IMU	BNO-055	Provides inertial measurements	USB link to Raspberry Pi
	2D Lidar	LDROBOT STL-19	Environment scanning and obstacle detection	USB link to Raspberry Pi
	RGB Camera	Logitech C922 Pro HD	Future integration with vision-based systems	USB link to Raspberry Pi
Power	Battery	12 V NiMH Nested Battery	Powers the robot	N/A

**Table 2:** Real-world spatial error statistics by dynamics (AI-enabled).

Dynamics	Error_x Mean (m)	Error_x Std	Error_x Min	Error_x Max	Error_y Mean (m)	Error_y Std	Error_y Min	Error_y Max
Static	−0.0021	0.0892	−0.3095	0.1295	0.0526	0.0926	−0.0598	0.2325
Moderate	−0.0570	0.0935	−0.3095	0.1275	0.0573	0.0790	−0.0562	0.2325
Aggressive	−0.1596	0.0829	−0.3095	0.1075	0.0927	0.0825	−0.0359	0.2238

**Table 3:** Real-world yaw prediction error |*error_yaw* − *yaw_diff*| by dynamics (AI-enabled).

Dynamics	|Error_Yaw − Yaw_Diff| Mean (rad)	Std	Min	Max
Static	0.0543	0.0899	0	0.9491
Moderate	0.0710	0.0975	0.0001	0.6680
Aggressive	0.2257	0.2600	0.0005	0.9491

## Data Availability

The code and data supporting this study are available in the following repositories: ROS_ESP32_Bridge (https://github.com/bogdan-abaza/ROS_ESP32_Bridge) accessed on 14 April 2025 and ai_tools (https://github.com/bogdan-abaza/ai_tools), accessed on 14 April 2025.

## References

[1] Macenski S., Foote T., Gerkey B., Lalancette G., Woodall W. (2022). Robot Operating System 2: Design, architecture, and uses in the wild. Sci. Robot..

[2] Macenski S., Soragna A., Carroll M., Ge Z. (2023). Impact of ROS 2 Node Composition in Robotic Systems. IEEE Robot. Auton. Lett. (RA-L).

[3] Moore T., Stouch D., Menegatti E., Michael N., Berns K., Yamaguchi H. (2003). A Generalized Extended Kalman Filter Implementation for the Robot Operating System. Intelligent Autonomous Systems 13.

[4] robot_localization. ROS Index. https://index.ros.org/r/robot_localization/.

[5] robot_localization—State Estimation Nodes. ROS Documentation. https://docs.ros.org/en/melodic/api/robot_localization/html/state_estimation_nodes.html.

[6] robot_localization—Sensor Configuration. ROS Documentation. https://docs.ros.org/en/melodic/api/robot_localization/html/configuring_robot_localization.html#sensor-configuration.

[7] REP-105: Coordinate Frames for Mobile Platforms. ROS Enhancement Proposals. https://www.ros.org/reps/rep-0105.html.

[8] Jugravu B.A., Lazăr M.V., Savu T., Abaza B.F., Opran C.G. (2024). Research on Position Accuracy of Autonomous Industrial Vehicles in Polymer Products Factories. Macromol. Symp..

[9] Eang C., Lee S. (2024). An Integration of Deep Neural Network-Based Extended Kalman Filter (DNN-EKF) Method in Ultra-Wideband (UWB) Localization for Distance Loss Optimization. Sensors.

[10] Luo L., Peng F., Dong L. (2024). Improved Multi-Sensor Fusion Dynamic Odometry Based on Neural Networks. Sensors.

[11] Djedili A., Amouri A., Laib Dit Leksir Y., Belkhiri A. (2024). Performance Evaluation of Population-Based Metaheuristic Algorithms for Solving the Inverse Kinematic of a Class of Continuum Robots. Sci. Bull. Univ. Politeh. Buchar. Ser. D.

[12] Kendall A., Grimes M., Cipolla R. PoseNet: A Convolutional Network for Real-Time 6-DOF Camera Relocalization. Proceedings of the IEEE International Conference on Computer Vision (ICCV).

[13] Chen R., Karl J., Ma H., Garg A., Atanasov N. Learning Uncertainty-Aware Dynamics Models Using Particle Filtering and Deep Ensembles. Proceedings of the Robotics: Science and Systems (RSS).

[14] Macenski S., Moore T., Lu D., Merzlyakov A., Ferguson M. (2023). From the Desks of ROS Maintainers: A Survey of Modern & Capable Mobile Robotics Algorithms in the Robot Operating System 2. Robot. Auton. Syst..

[15] Ye Y., Nie Z., Liu X., Xie F., Li Z., Li P. (2023). ROS2 Real-time Performance Optimization and Evaluation. Chin. J. Mech. Eng..

[16] Hachem M., Borrell A.M., Sename O., Atoui H., Morato M. (2023). ROS Implementation of Planning and Robust Control Strategies for Autonomous Vehicles. Electronics.

[17] Won H.M., Lee J., Oh J. (2024). Localization Meets Uncertainty: Uncertainty-Aware Multi-Modal Localization. arXiv.

[18] Wang R., Sun W., He Y., Han F., Zhang Y. End-to-End Deep Learning of Visual Odometry with Uncertainty Estimation. Proceedings of the IEEE/RSJ International Conference on Intelligent Robots and Systems (IROS).

[19] Abaza B., Paraschiv I., Spiroiu M., Stanciu C. (2015). Project-based pedagogy for a new product development. Proceedings of the 9th International Technology, Education and Development Conference, INTED 2015.

[20] ROS 2 diff_drive_controller Documentation. ROS Control Documentation. https://control.ros.org/humble/doc/ros2_controllers/diff_drive_controller/doc/userdoc.html.

[21] Alqahtani D.K., Cheema A., Toosi A.N. (2024). Benchmarking Deep Learning Models for Object Detection on Edge Computing Devices. arXiv.

[22] Hochreiter S., Schmidhuber J. (1997). Long Short-Term Memory. Neural Comput..

[23] Rasmussen C.E., Williams C.K.I. (2006). Gaussian Processes for Machine Learning.

[24] Ke G., Meng Q., Finley T., Wang T., Chen W., Ma W., Ye Q., Liu T.Y. (2017). LightGBM: A Highly Efficient Gradient Boosting Decision Tree. Adv. Neural Inf. Process. Syst..

[25] Ameen S., Varghese S. (2024). Efficient Convolutional Neural Networks on Raspberry Pi: Enhancing Performance with Pruning and Quantization. Research Square.

[26] Pedregosa F., Varoquaux G., Gramfort A., Michel V., Thirion B., Grisel O., Blondel M., Prettenhofer P., Weiss R., Dubourg V. (2011). Scikit-learn: Machine Learning in Python. J. Mach. Learn. Res..

